# Association Between Idiopathic Intracranial Hypertension and Risk of Cardiovascular Diseases in Women in the United Kingdom

**DOI:** 10.1001/jamaneurol.2019.1812

**Published:** 2019-07-08

**Authors:** Nicola J. Adderley, Anuradhaa Subramanian, Krishnarajah Nirantharakumar, Andreas Yiangou, Krishna M. Gokhale, Susan P. Mollan, Alexandra J. Sinclair

**Affiliations:** 1 Institute of Applied Health Research, University of Birmingham, Birmingham, United Kingdom; 2 Centre for Endocrinology, Diabetes, and Metabolism, Birmingham Health Partners, Birmingham, United Kingdom; 3 Health Data Research UK, Birmingham, United Kingdom; 4 Metabolic Neurology, Institute of Metabolism and Systems Research, University of Birmingham, Birmingham, United Kingdom; 5 Department of Neurology, University Hospitals Birmingham, Queen Elizabeth Hospital, Birmingham, United Kingdom; 6 Birmingham Neuro-Ophthalmology, Queen Elizabeth Hospital, Birmingham, United Kingdom

## Abstract

**Importance:**

Cardiovascular disease (CVD) risk has not been previously evaluated in a large matched cohort study in idiopathic intracranial hypertension (IIH).

**Objectives:**

To estimate the risk of composite cardiovascular events, heart failure, ischemic heart disease, stroke/transient ischemic attack (TIA), type 2 diabetes, and hypertension in women with idiopathic intracranial hypertension and compare it with the risk in women, matched on body mass index (BMI) and age, without the condition; and to evaluate the prevalence and incidence of IIH.

**Design, Setting, and Participants:**

This population-based matched controlled cohort study used 28 years of data, from January 1, 1990, to January 17, 2018, from The Health Improvement Network (THIN), an anonymized, nationally representative electronic medical records database in the United Kingdom. All female patients aged 16 years or older were eligible for inclusion. Female patients with IIH (n = 2760) were included and randomly matched with up to 10 control patients (n = 27 125) by BMI and age.

**Main Outcomes and Measures:**

Adjusted hazard ratios (aHRs) of cardiovascular outcomes were calculated using Cox regression models. The primary outcome was a composite of any CVD (heart failure, ischemic heart disease, and stroke/TIA), and the secondary outcomes were each CVD outcome, type 2 diabetes, and hypertension.

**Results:**

In total, 2760 women with IIH and 27 125 women without IIH were included. Age and BMI were similar between the 2 groups, with a median (interquartile range) age of 32.1 (25.6-42.0) years in the exposed group and 32.1 (25.7-42.1) years in the control group; in the exposed group 1728 women (62.6%) were obese, and in the control group 16514 women (60.9%) were obese. Higher absolute risks for all cardiovascular outcomes were observed in women with IIH compared with control patients. The aHRs were as follows: composite cardiovascular events, 2.10 (95% CI, 1.61-2.74; *P* < .001); heart failure, 1.97 (95% CI, 1.16-3.37; *P* = .01); ischemic heart disease, 1.94 (95% CI, 1.27-2.94; *P* = .002); stroke/TIA, 2.27 (95% CI, 1.61-3.21; *P* < .001); type 2 diabetes, 1.30 (95% CI, 1.07-1.57; *P* = .009); and hypertension, 1.55 (95% CI, 1.30-1.84; *P* < .001). The incidence of IIH in female patients more than tripled between 2005 and 2017, from 2.5 to 9.3 per 100 000 person-years. Similarly, IIH prevalence increased in the same period, from 26 to 79 per 100 000 women. Incidence increased markedly with BMI higher than 30.

**Conclusions and Relevance:**

Idiopathic intracranial hypertension in women appeared to be associated with a 2-fold increase in CVD risk; change in patient care to modify risk factors for CVD may reduce long-term morbidity for women with IIH and warrants further evaluation.

## Key Points

**Question:**

Is the risk of cardiovascular disease greater in women with idiopathic intracranial hypertension than in women of the same age and body mass index but without idiopathic intracranial hypertension?

**Findings:**

In this population-based matched controlled cohort study of 2760 female patients with idiopathic intracranial hypertension and 27 125 control patients, women with this condition had twice the risk for cardiovascular disease compared with their counterparts with similar body mass index and age. Between 2005 and 2017, the incidence and prevalence of idiopathic intracranial hypertension have tripled.

**Meaning:**

Idiopathic intracranial hypertension appeared to be a risk factor for cardiovascular disease in women; changing patient management to address the risk factors for cardiovascular disease may reduce long-term morbidity.

## Teaser

This study analyzes the electronic medical records stored in The Health Improvement Network database of UK women with idiopathic intracranial hypertension to identify their cardiometabolic risks and outcomes.

## Introduction

Idiopathic intracranial hypertension (IIH) is a condition of unknown origin characterized by elevated intracranial pressure typically manifesting as papilledema, with a consequent risk of visual loss and chronic disabling headache.^1,2^ Incidence and economic burden of IIH are rising in line with global obesity figures.^3^ Diagnostic criteria include brain imaging to exclude mass lesion, hydrocephalus, and venous thrombosis as well as the presence of papilledema and a lumbar puncture pressure greater than 25 cm cerebrospinal fluid with normal constituents.^4^ Currently, management focuses on preserving vision and reducing headache morbidity.^5^

Patients with IIH are typically female, and more than 90% of them are obese.^6^ Body mass index (BMI; calculated as weight in kilograms divided by height in meters squared) is associated with the risk of IIH.^7^ Obesity is an established risk factor for cardiovascular disease (CVD)^8^ and type 2 diabetes.^9,10^ Obesity alone may confer a higher risk of CVD in IIH. Idiopathic intracranial hypertension is characterized by a unique profile of androgen excess affecting the cerebrospinal fluid dynamics.^11^ Androgen excess is a key driver of increased cardiovascular risk.^12,13,14,15,16,17^ We hypothesized that patients with IIH may have an increased risk of cardiometabolic disease compared with a cohort matched for obesity (BMI), age, and sex. The aim of this study was to define cardiometabolic risk in IIH, independent of BMI, and to identify trends in prevalence and incidence.

## Methods

### Design and Data Source

Yearly prevalence was estimated by performing sequential cross-sectional studies on January 1 each calendar year from 2005 to 2017. Annual IIH incidence rates for the same period were identified by conducting a series of yearly cohort studies. Long-term cardiometabolic outcomes were assessed through a retrospective population-based matched controlled cohort study using data, dated January 1, 1990, to January 17, 2018, from The Health Improvement Network (THIN). The THIN data-collection scheme and research using THIN data were approved by the NHS South-East Multicenter Research Ethics Committee in 2003. Under the terms of this approval, studies must undergo independent scientific review. Approval for the use of THIN data in this present analysis was obtained from the Scientific Review Committee in 2018. Because THIN data are anonymized, individual patients are not required to give consent for the use of their data.

The Health Improvement Network is a national database of electronic primary care records generalizable to the UK population. It contains coded information for more than 15 million patients from 787 primary care general practices, including patient demographics, symptoms, diagnoses, drug prescriptions, consultations, and laboratory test results. Cardiovascular diseases, diabetes, and hypertension are included in the Quality and Outcomes Framework, a scheme that incentivizes appropriate identification and management of patients with these diagnoses.^18^ Therefore, these conditions are well coded in primary care and have been extensively studied (eAppendix 1 in the Supplement).^19,20^

### Study Population 

General practices were eligible for inclusion 1 year after reporting acceptable mortality rates (a measure of data quality) and 1 year after starting to use electronic medical records (to ensure sufficient time for recording of comorbidities). For incidence and prevalence, female patients of all ages who registered with an eligible general practice for 1 year or more before cohort entry (to ensure documentation of all important baseline covariates) were eligible for inclusion. In the cardiometabolic outcomes cohort, only females aged 16 years or older were included.

An IIH diagnosis was indicated by a record of a relevant clinical (Read) code. In the United Kingdom, IIH is diagnosed by hospital specialists according to diagnostic criteria^4^ and is then communicated to general practices in which the clinical code is entered into the patient’s electronic medical record. Patients with a clinical code for IIH and a simultaneous code for hydrocephalus or cerebral venous thrombosis were excluded to avoid the risk of miscoding.

In the cardiometabolic outcomes cohort study, for each patient with IIH (exposed group), up to 10 controls were randomly selected from a pool of age-, sex-, and BMI-matched patients. Age was matched to within 1 year, and BMI was matched to within 2 kg/m^2^. Matched control patients were selected with this method: Patients with IIH were identified, and their order was shuffled by randomly permuting the patient list (following the Fisher Yates algorithm) using a linear congruential generator as the source of randomness. Shuffling ensured that all patients in the exposed group had an equal chance of being matched to a control in instances in which a control individual could potentially be matched with more than 1 exposed patient. When the required number (10) of possible control patients for a particular exposed patient was exceeded, a linear congruential generator provided a random number between 1 and the number of potential control patients. The potential control at the position of this random number was selected. This process was repeated for subsequent control patients.

For patients with a new IIH diagnosis, the index date was the date of diagnosis. For patients with a preexisting diagnosis, the index date was 1 year after the registration date. Control patients were assigned the same index date as their corresponding exposed patient to avoid immortal time bias.^21^ Patients with IIH and control patients were followed up from the index date until the earliest of these dates: outcome event, death, patient left the general practice, general practice ceased contributing to the database, or study end.

The primary outcome was a composite of any CVD: heart failure, ischemic heart disease (IHD), and stroke or transient ischemic attack (TIA). Secondary outcomes were the cardiovascular outcomes separately, type 2 diabetes, and hypertension. Patients with a record of the outcome of interest at baseline were excluded from the corresponding analysis; for CVD, patients with a record of any CVD at baseline were excluded; for type 2 diabetes, patients with a record of type 1 or type 2 diabetes at baseline were excluded.

Outcomes were defined by clinical codes. Ischemic heart disease was defined in accordance with the Quality and Outcomes Framework^22^ definition of coronary heart disease, which included myocardial infarction, angina, coronary atherosclerosis, ischemic cardiomyopathy, and coronary microvascular disease.

Covariates that might be associated with outcomes were selected on the basis of biological plausibility and use in previous research. Covariates considered for all outcomes included age, BMI, social deprivation, smoking status, current lipid-lowering medication prescription, baseline record of migraine, and baseline cardiometabolic conditions (excluding the outcome). In the event BMI was not available at baseline, a BMI recorded up to 90 days after the index date was used. Body mass index was categorized as lower than 25 (underweight to healthy weight), 25-30 (overweight), and higher than 30 (obese). Social deprivation was recorded as Townsend deprivation quintile (scale: 1-5, with 1 indicating least deprived).^23^ Smoking status was categorized as nonsmoker, smoker, and ex-smoker. Comorbidities were defined using relevant clinical codes.

### Statistical Analysis

For point prevalence, the proportion of eligible female patients with any record ever of IIH was calculated on January 1 each year from 2005 to 2017. Crude incidence rates of IIH for every year from 2005 to 2017 were calculated by dividing the number of female patients with new IIH diagnosis (numerator) by the total number of person-years at risk (denominator) for the given year. To describe incidence rates stratified by age at diagnosis, baseline BMI, and Townsend deprivation quintile, we used data for the whole period (2005-2017). To estimate the independent association of age at diagnosis, BMI, and Townsend deprivation quintile with IIH incidence, we calculated adjusted incidence rate ratios using Poisson regression, offsetting for person-years of follow-up. For the missing data for BMI and Townsend deprivation quintile, a separate missing category was created, which was included in the regression analysis; BMI value was categorized as missing if no BMI was recorded at baseline or within 90 days of the index date, or if the recorded value was outside the plausible range (14-75).

In the cardiometabolic outcomes cohort study, crude hazard ratios (HRs) and adjusted HRs (aHR) and their corresponding 95% CIs were calculated using Cox proportional hazards regression models. The proportional hazards assumption was checked using log-log plots and the Schoenfeld residuals test. For the primary outcome (composite CVD) and each of the secondary outcomes (heart failure, IHD, stroke/TIA, diabetes, and hypertension), 3 nested adjusted Cox models were assessed. Model 1 was adjusted for the baseline patient demographic variables: age, BMI (continuous), Townsend deprivation quintile, and smoking status. Model 2 was adjusted for baseline demographic variables, current (up to 65 days before the index date) lipid-lowering drug prescription, and baseline cardiometabolic comorbidities (for cardiovascular outcomes: hypertension and diabetes; for diabetes outcome: heart failure, IHD, stroke/TIA, and hypertension; and for hypertension outcome: heart failure, IHD, stroke/TIA, and diabetes). Model 3 included all model 2 covariates plus migraine. The Nelson-Aalen cumulative hazard function was used to plot cumulative hazard of primary and secondary outcomes.

In primary analysis, all prevalent and incident IIH diagnoses were included. Because IIH is a rare disease, all female patients with a diagnosis of IIH were included to maximize the sample size. However, a sensitivity analysis limited to patients with incident IIH (newly diagnosed after registration with the general practice) and the corresponding control patients was performed to explore any association with survival bias. Another sensitivity analysis limited to women diagnosed with IIH at age 60 years or younger was carried out, because IIH is rare among older adults and misclassification error may be present in those diagnosed older than age 60 years. Sensitivity analyses were adjusted for covariates in model 2.

Body mass index was treated as a continuous variable. Missing BMI data were replaced using multiple imputation. Multiple imputation was performed using chained equations and predictive mean matching. Values were imputed using the following covariates: age; Townsend deprivation quintile; smoking status; baseline diabetes, hypertension, heart failure, IHD, and stroke/TIA; and current lipid-lowering drug prescription.

Baseline covariates were summarized for both exposed and control groups using appropriate descriptive statistics: mean (SD) and median (interquartile range [IQR]) for continuous variables, and number (%) for categorical variables. All analyses were performed with Stata IC, version 14 (StataCorp LLC). Two-sided, unpaired *P* values were calculated using the *t* test. Statistical significance was set at *P* < .05.

## Results

**Figure 1.**
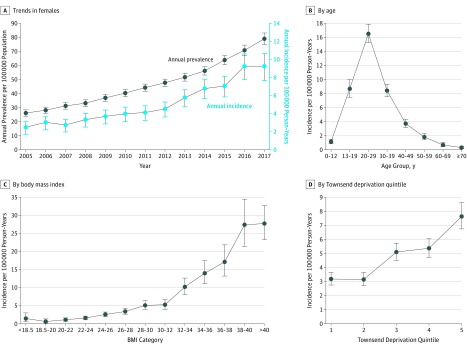
Prevalence and Incidence of Idiopathic Intracranial Hypertension in Female Patients, 2005-2017 Body mass index is calculated as weight in kilograms divided by height in meters squared. Townsend deprivation quintile scale: 1-5, with 1 being least deprived and 5 most deprived.

Prevalence of IIH in female patients increased from 26 per 100 000 in 2005 to 79 per 100 000 in 2017 (Figure 1A, eTable 1 in the Supplement). Annual incidence of IIH in female patients increased from 2.5 per 100 000 person-years in 2005 to 9.3 per 100 000 person-years in 2017 (Figure 1A). Incidence was highest in the 20- to 29-year age group (16.5 per 100 000 person-years) followed by the 13- to 19-year age group (8.7 per 100 000 person-years) and the 30- to 39-year age group (8.4 per 100 000 person-years) (Figure 1B). Incidence of IIH increased with increasing BMI (Figure 1C). After adjustments for age at diagnosis and Townsend deprivation quintile, the incidence rate ratio for IIH among patients with BMI of 25 to 30 was 3.76 (95% CI, 2.98-4.74; *P* < .001) and with BMI higher than 30 was 17.55 (95% CI, 14.41-21.36; *P* < .001), compared with patients with BMI lower than 25 (eTable 2 in the Supplement). Incidence of IIH also appeared to be higher among female patients who were in lower socioeconomic quintiles (Figure 1D). However, after adjustments for age and BMI, the trend was lost. The most-deprived Townsend quintile remained statistically significantly associated with the increased risk of IIH among female patients (incidence rate ratio, 1.46; 95% CI, 1.22-1.77; *P* < .001).

In total, 2760 women with IIH (9.2%) and 27 125 women without IIH (90.8%), matched for age and BMI, were included in the analyses investigating cardiometabolic outcomes. The median (IQR) follow-up time was 3.5 (1.3-7.5) years. Baseline characteristics of female patients with IIH (exposed group) and without IIH (control group) are presented in Table 1. At cohort entry, age and BMI were similar between the 2 groups, with a median (IQR) age of 32.1 (25.6-42.0) years in the exposed group and 32.1 (25.7-42.1) years in the control group; in the exposed group 1728 women (62.6%) were obese, and in the control group 16 514 women (60.9%) were obese. Women with IIH, compared with those without, were slightly more likely to be smokers (849 [30.8%] vs 6134 [22.6%]) or ex-smokers (502 [18.2%] vs 4573 [16.9%]). Women with IIH had substantially higher migraine, CVD, and hypertension at baseline compared with female patients without IIH.

**Table 1.** Baseline Characteristics of Women With or Without Idiopathic Intracranial Hypertension

Variable	No. (%)	
	Women With IIH (Exposed Group)	Women Without IIH (Control Group)
Population	2760 (9.2)	27 125 (90.8)
Incident cohort	1331 (48.2)	12 679 (46.7)
Population aged <60 y	2709 (98.1)	25 811 (95.2)
Age, median (IQR), y	32.1 (25.62-42.00)	32.1 (25.71-42.06)
Townsend deprivation quintile		
1 (Least deprived)	361 (13.1)	4268 (15.7)
2	381 (13.8)	4397 (16.2)
3	532 (19.3)	5174 (19.1)
4	538 (19.5)	5122 (18.9)
5 (Most deprived)	454 (16.5)	4134 (15.2)
Missing data	494 (17.9)	4030 (14.9)
Smoking status		
Nonsmoker	1284 (46.5)	15 058 (55.5)
Ex-smoker	502 (18.2)	4573 (16.9)
Smoker	849 (30.8)	6134 (22.6)
Missing data	125 (4.5)	1360 (5.0)
BMI, median (IQR)	34.80 (29.30-40.30)	34.30 (29.00-39.70)
BMI		
<25	246 (8.9)	2561 (9.4)
25-30	416 (15.1)	4203 (15.5)
>30	1728 (62.6)	16 514 (60.9)
Missing data	370 (13.4)	3847 (14.2)
Current lipid prescription	180 (6.5)	1572 (5.8)
Migraine	580 (21.0)	3247 (11.9)
Outcomes at baseline		
Heart failure	8 (0.3)	58 (0.2)
IHD	35 (1.3)	245 (0.9)
Stroke/TIA	46 (1.7)	189 (0.7)
Hypertension	380 (13.8)	2500 (9.2)
Type 2 diabetes	130 (4.7)	1425 (5.2)

Abbreviations: BMI, body mass index (calculated as weight in kilograms divided by height in meters squared); IHD, ischemic heart disease; IIH, idiopathic intracranial hypertension; IQR, interquartile range; TIA, transient ischemic attack.

**Figure 2.**
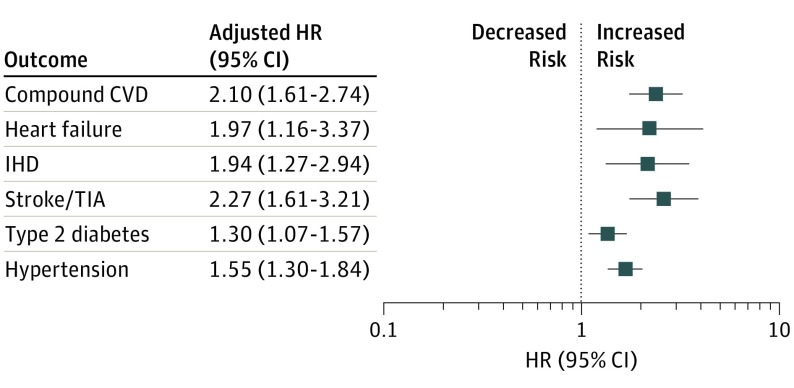
Composite Outcomes Among Women With or Without Idiopathic Intracranial Hypertension CVD indicates cardiovascular disease; HR, hazard ratio; IHD, ischemic heart disease; and TIA, transient ischemic attack.

**Figure 3.**
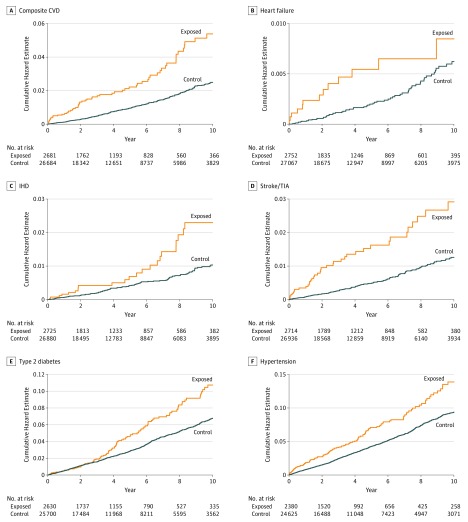
Cardiometabolic Outcomes in Women With (Exposed) or Without (Control) Idiopathic Intracranial Hypertension CVD, cardiovascular disease; IHD, ischemic heart disease; and TIA, transient ischemic attack.

Crude incidence rate of composite cardiovascular events was 5.31 per 1000 person-years among women in the exposed group, with a crude HR of 2.15 (95% CI, 1.66-2.79; *P* < .001), and was 2.47 per 1000 person-years among women in the control group. Adjusting for baseline demographic variables and cardiovascular risk factors (model 2) marginally reduced the effect estimate (aHR, 2.10; 95% CI, 1.61-2.74; *P* < .001) (Table 2; Figure 2 and Figure 3). Further adjusting for baseline diagnosis of migraine (model 3) was not associated with any changes in the results.

**Table 2.** Incidence Rates and Hazard Ratios for Cardiometabolic Outcomes in Women With or Without Idiopathic Intracranial Hypertension

Outcome	Women With IIH (Exposed Group)	*P* Value	Women Without IIH (Control Group)
**Composite CVD**			
Population, No.	2613	NA	26 356
Outcome events, No. (%)	68 (2.5)	NA	328 (1.2)
Person-years	12 809	NA	132 930
Crude incidence rate per 1000 person-years	5.31	NA	2.47
Follow-up, median (IQR), y	3.50 (1.34-7.11)	NA	3.72 (1.51-7.39)
Crude HR (95% CI)	2.15 (1.66-2.79)	<.001	NA
Adjusted HR (95% CI)			
Model 1^a^	2.10 (1.61-2.73)	<.001	NA
Model 2^b^	2.10 (1.61-2.74)	<.001	NA
Model 3^c^	2.08 (1.59-2.71)	<.001	NA
**Heart Failure**			
Population, No.	2735	NA	26 989
Outcome events, No. (%)	17 (0.6)	NA	78 (0.3)
Person-years	13 445	NA	136 357
Crude incidence rate per 1000 person-years	1.26	NA	0.57
Follow-up, median (IQR), y	3.58 (1.38-7.26)	NA	3.77 (1.52-7.50)
Crude HR (95% CI)	2.19 (1.30-3.71)	.003	NA
Adjusted HR (95% CI)			
Model 1^a^	1.99 (1.17-3.37)	.01	NA
Model 2^b^	1.97 (1.16-3.37)	.01	NA
Model 3^c^	1.97 (1.15-3.36)	.01	NA
**Ischemic Heart Disease**			
Population, No.	2698	NA	26 749
Outcome events, No. (%)	27 (0.9)	NA	131 (0.5)
Person-years	13 216	NA	134 521
Crude incidence rate per 1000 person-years	2.04	NA	0.97
Follow-up, median (IQR), y	3.56 (1.37-7.20)	NA	3.73 (1.51-7.42)
Crude HR (95% CI)	2.10 (1.39-3.18)	<.001	NA
Adjusted HR (95% CI)			
Model 1^a^	1.92 (1.27-2.91)	.002	NA
Model 2^b^	1.94 (1.27-2.94)	.002	NA
Model 3^c^	1.89 (1.24-2.88)	.003	NA
**Stroke/TIA**			
Population, No.	2674	NA	26 755
Outcome events, No. (%)	40 (1.5)	NA	181 (0.7)
Person-years	13 115	NA	135 271
Crude incidence rate per 1000 person-years	3.05	NA	1.34
Follow-up, median (IQR), y	3.51 (1.34-7.17)	NA	3.76 (1.52-7.47)
Crude HR (95% CI)	2.27 (1.61-3.19)	<.001	NA
Adjusted HR (95% CI)			
Model 1^a^	2.27 (1.61-3.21)	<.001	NA
Model 2^b^	2.27 (1.61-3.21)	<.001	NA
Model 3^c^	2.24 (1.58-3.17)	<.001	NA
**Hypertension**			
Population, No.	2232	NA	23 566
Outcome events, No. (%)	148 (6.2)	NA	1059 (4.3)
Person-years	10 505	NA	115 800
Crude incidence rate per 1000 person-years	14.09	NA	9.15
Follow-up, median (IQR), y	3.20 (1.26-6.40)	NA	3.48 (1.43-6.94)
Crude HR (95% CI)	1.55 (1.30-1.84)	<.001	NA
Adjusted HR (95% CI)			
Model 1^a^	1.53 (1.29-1.82)	<.001	NA
Model 2^d^	1.55 (1.30-1.84)	<.001	NA
Model 3^c^	1.55 (1.30-1.84)	<.001	NA
**Type 2 Diabetes**			
Population, No.	2510	NA	24 901
Outcome events, No. (%)	120 (4.6)	NA	799 (3.1)
Person-years	12 300	NA	125 947
Crude incidence rate per 1000 person-years	9.76	NA	6.34
Follow-up, median (IQR), y	3.49 (1.34-6.94)	NA	3.62 (1.47-7.27)
Crude HR (95% CI)	1.55 (1.28-1.87)	<.001	NA
Adjusted HR (95% CI)			
Model 1^a^	1.32 (1.09-1.61)	.005	NA
Model 2^e^	1.30 (1.07-1.57)	.009	NA
Model 3^c^	1.29 (1.06-1.57)	.01	NA

Abbreviations: BMI, body mass index (calculated as weight in kilograms divided by height in meters squared); CVD, cardiovascular disease; HR, hazard ratio; IHD, ischemic heart disease; IIH, idiopathic intracranial hypertension; IQR, interquartile range; NA, not applicable; TIA, transient ischemic attack.

^a^ Model 1 adjusted for age, BMI (continuous), Townsend deprivation quintile (scale: 1-5, with 1 indicating least deprived), and smoking status.

^b^ Model 2 adjusted for all covariates in model 1 plus current (up to 65 days prior to index date) lipid-lowering drug prescription and baseline hypertension and diabetes.

^c^ Model 3 included all model 2 covariates plus migraine.

^d^ Model 2 adjusted for all covariates in model 1 plus current (up to 65 days prior to index date) lipid-lowering drug prescription and baseline IHD, heart failure, stroke/TIA, and hypertension.

^e^ Model 2 adjusted for all covariates in model 1 plus current (up to 65 days prior to index date) lipid-lowering drug prescription and baseline IHD, heart failure, stroke/TIA, and diabetes.

All adjusted HRs (model 2) for individual CVD outcomes showed a statistically significant increase in risk in patients with IIH: heart failure, 1.97 (95% CI, 1.16-3.37; *P* = .01); IHD, 1.94 (95% CI, 1.27-2.94; *P* = .002); and stroke/TIA, 2.27 (95% CI, 1.61-3.21; *P* < .001).

The association between IIH and composite cardiovascular events remained statistically significant, with a similar effect size, in the 2 sensitivity analyses performed (adjusted for covariates in model 2). The HR from the sensitivity analysis restricted to patients with incident (new diagnosis) IIH and the corresponding control female patients was 3.14 (95% CI, 2.07-4.78; *P* < .001). The HR from the analysis restricted to women who received a diagnosis before 60 years of age was 2.09 (95% CI, 1.58-2.75; *P* < .001).

For type 2 diabetes, the crude incidence rate was 9.8 per 1000 person-years in women with IIH and 6.3 per 1000 person-years in those without IIH. The crude HR was 1.55 (95% CI, 1.28-1.87; *P* < .001). Adjusting for baseline demographic variables and comorbidities was associated with a slight reduction in the effect estimate (aHR, 1.30; 95% CI, 1.07-1.57; *P* = .009) (Table 2; Figures 2 and 3).

For hypertension, the crude incidence rate was 14.1 per 1000 person-years in women with IIH and 9.2 per 1000 person-years in those without IIH. The crude HR was 1.55 (95% CI, 1.30-1.84; *P* < .001), and adjusting for baseline demographic variables and comorbidities was not associated with any changes.

### Sensitivity Analyses

In sensitivity analyses restricted to patients with incident (newly diagnosed) IIH and their corresponding controls, the median (IQR) age was 29.6 (24.2-38.2) years, and the median (IQR) follow-up was 3.1 (1.3-6.1) years. The association between IIH and composite cardiovascular events remained statistically significant, with a similar effect size (aHR, 3.14; 95% CI, 2.07-4.78; *P* < .001). The association with type 2 diabetes was lost (aHR, 0.82; 95% CI, 0.57-1.18; *P* = .28), and the association with hypertension remained statistically significant with a slight increase in effect estimate (aHR, 1.95; 95% CI, 1.49-2.56; *P* < .001) (eTable 3 in the Supplement).

In sensitivity analyses restricted to women who received an IIH diagnosis before 60 years of age, the aHRs remained similar to those in the primary analyses: composite CVD, 2.09 (95% CI, 1.58-2.75; *P* < .001); diabetes, 1.35 (95% CI, 1.10-1.64; *P* = .003); and hypertension, 1.60 (95% CI, 1.34-1.92; *P* < .001) (eTable 3 in the Supplement).

## Discussion

In this population-based matched controlled cohort study, women with IIH had more than twice the risk of cardiovascular events compared with control patients without IIH matched for BMI and age. Absolute risk for this young population was low but noteworthy, considering the young age of the sample and the relatively short median follow-up (approximately 3½ years). The disease burden of IIH is growing, and its prevalence and incidence are rising annually and heightened in those with obesity.

To our knowledge, no previous cohort study has evaluated cardiovascular risk in IIH.^24^ This current study has identified that an IIH diagnosis is statistically significantly associated with increased risk of composite cardiovascular events, heart failure, IHD, and stroke/TIA, independent of BMI. This finding is the first indication that morbidity in IIH may extend beyond the typically considered areas of visual loss and chronic headaches.^1,2,6,25^ The predominant occurrence of obesity in IIH may be expected to increase the cardiovascular risk in these patients; however, these findings are apparent despite matching for BMI and age and controlling for important covariates.

Previous evidence has suggested an association between migraine and increased risk of CVD.^26^ Migraine is diagnosed in approximately 80% of patients with IIH.^27^ Therefore, we included migraine as a covariate in model 3 to assess whether comorbid migraine was a factor in the observed increase in CVD risk; however, the results were unaffected, suggesting no association.

Idiopathic intracranial hypertension was associated with a 30% increase in risk of diabetes and a 55% increase in risk of hypertension in women, independent of BMI and age and after accounting for potential confounders. In a sensitivity analysis limited to female patients with new IIH diagnosis, the association with diabetes was lost. The reason for this lost association may be the younger age of patients with incident IIH (median age of 29 years) and the relatively short follow-up (median length of 3 years), given that onset and diagnosis of type 2 diabetes typically occur at an older age (as reflected in the cumulative hazard plot [Figure 3]).

The underlying mechanisms of the increased cardiovascular risk in IIH are not established. Higher cardiovascular risk would be estimated in an obese population, but it was observed despite matching to an obese control population. This finding adds support to the concept that cardiovascular risk in IIH is not exclusively associated with obesity, and it points to a more complex disease characterized by systemic metabolic dysregulation. Centripetal fat accumulation is key in determining the risk of metabolic syndrome and CVD.^28,29,30^ Centripetal fat predominance has been identified in IIH and correlates with intracranial pressure.^31^ Whether obesity is causal in IIH, or if it represents a comorbidity driven by the underlying metabolic disease state, is unclear. However, weight loss is associated with resolution of IIH.^32^ Evaluating the association of weight modification with cardiovascular risk in IIH is of future interest.

To date, IIH has been considered a rare condition, with reported incidence of 1 to 3 per 100 000 in the general population, but IIH incidence has been speculated to be rising, secondary to the obesity epidemic that has been spreading globally over the past 40 years.^1,33^ In this large cohort study, recorded prevalence and incidence of IIH tripled between 2005 and 2017, mirroring other reports.^3,34^ Incidence is highest in female patients aged 20 to 29 years, with few patients older than 50 years receiving a diagnosis. The condition is much more frequent in those who are overweight and obese, and the adjusted incidence increases 18-fold in those with a BMI higher than 30, compared with those with a BMI in the healthy weight range. Incidence rises dramatically with a BMI of 30 or higher. Although the rising IIH incidence rates are in line with growing obesity rates, the numbers could also represent increased reporting of this condition. However, cerebrospinal fluid shunt surgery for IIH has also increased dramatically (320% increase in surgical procedures between 1988 and 2002).^35^

Idiopathic intracranial hypertension occurrence increased with higher Townsend deprivation levels. The association between social deprivation and increased morbidity and mortality has been well established.^36,37,38,39,40^ The association between increased incidence of IIH and the most-deprived quintile remained after adjustments for age and BMI, suggesting that obesity may not be the only factor in higher incidence in the most deprived population.

### Implications

This study has demonstrated increased cardiovascular risk in patients with IIH, despite their young age and independent of BMI. These findings would support broadening the care for patients with IIH to include evaluating and modifying cardiovascular risk.

Patients with IIH are typically identified at a young age, which could provide the opportunity for early assessment for modifiable cardiovascular risk factors with subsequent management. Early intervention is likely to improve long-term cardiometabolic outcomes as noted in other diseases.^41,42^ Weight loss can modify cardiometabolic disease risk, and it has also been shown as a treatment for IIH.^32^ The role of bariatric surgery to modify IIH is being evaluated, but its association with cardiometabolic health in the context of IIH is also of interest.^43^ Future studies that comprehensively evaluate the clinical and financial implications of modifying cardiometabolic risk for IIH are important.

### Strengths and Limitations

This study has several strengths, including its large sample size used to evaluate a rare condition. In addition, the THIN primary care database is generalizable to the UK population and has been found to be reliable.^44,45^ However, as with any routinely collected data, the use of this database presents potential biases, such as incorrect or inconsistent coding and incomplete data. An IIH diagnosis necessitates a hospital encounter for the diagnostic investigations,^3^ and the diagnosis is then reported to general practice or primary care physicians; this process increases the likelihood that the diagnosis in the primary care electronic record is correct, but accuracy cannot be guaranteed. To ensure that IIH mimics were not analyzed, we excluded patients with hydrocephalus or cerebral venous sinus thrombosis and, in a sensitivity analysis, those who received a diagnosis after 60 years of age. We used BMI at or near the baseline date; however, weight may fluctuate over time, and BMI at baseline may not be the same as that at the time of IIH or outcome diagnosis for all patients. These results are limited by the relatively short median follow-up.

Headache pain may have reduced physical activity in the exposed cohort. Physical activity was poorly recorded in primary care, and therefore adjusting for this potential confounder in the regression models was not possible. However, most of the women in the exposed and control cohorts were obese; levels of physical activity are substantially lower in people with obesity,^46,47^ and therefore any further reduction in physical activity in the IIH cohort was likely to be small. Furthermore, we adjusted for other cardiometabolic risk factors, such as hypertension, which are also associated with physical activity levels.^48^ In addition, recent research has shown no difference in adipose tissue distribution^31^ or muscle mass, a marker of physical activity,^49,50,51^ between individuals with IIH and BMI-matched control patients (eAppendix 2 and the eFigure in the Supplement). However, differences in physical activity could have had an association with CVD outcomes.

These results may have been affected by surveillance bias from a greater number of contacts between patients with IIH and health care professionals; however, because of the serious, symptomatic nature of the outcomes considered, this association is likely to be small. Increased risk of CVD in patients with IIH may be partially mediated by exposure to drugs, such as nonsteroidal anti-inflammatory drugs, during the study period; we did not account for these medications in the study design.

## Conclusions

Women with IIH appeared to have increased risk of CVD compared with women without IIH who were matched for BMI and age. The elevated risk of CVD is over and above that expected from obesity alone, suggesting additional systemic metabolic factors unique to IIH. Absolute risk in IIH remains low but is important to know given the rising incidence of this condition and the relatively young age of the patients with IIH diagnosis, which provides an opportunity for intervention. A step change in clinical practice to include assessment and management of cardiovascular risk in IIH is likely to be advantageous in efforts to improve long-term health outcomes for patients and warrants further evaluation.

## Notes

Supplement. **eAppendix 1.** Methods **eAppendix 2.** Muscle Mass **eFigure.** Muscle Mass in Patients With IIH and BMI-Matched Controls Without IIH **eTable 1.** Prevalence and Incidence of IIH Among Females in the UK From 2005 to 2017 **eTable 2.** Incidence of IIH Among Females From 2005 to 2015 Stratified By Age At Diagnosis, BMI and Townsend Score **eTable 3.** Sensitivity Analyses
