# Supplementary material for: Association Between Idiopathic Intracranial Hypertension and Risk of Cardiovascular Diseases in Women in the United Kingdom
Source: JAMA Neurol. 2019 Jul 8;76(9):1088–98. doi: 10.1001/jamaneurol.2019.1812 (PMC6618853; doi:10.1001/jamaneurol.2019.1812)
Supplement: Supplement. — eAppendix 1. Methods eAppendix 2. Muscle Mass eFigure. Muscle Mass in Patients With IIH and BMI-Matched Controls Without IIH eTable 1. Prevalence and Incidence of IIH Among Females in the UK From 2005 to 2017 eTable 2. Incidence of IIH Among Females From 2005 to 2015 Stratified By Age At Diagnosis, BMI and Townsend Score eTable 3. Sensitivity Analyses [file jamaneurol-76-1088-s001.pdf]

Supplementary Online Content

Adderley NJ, Subramanian A, Nirantharakumar K, et al. Association between idiopathic intracranial hypertension and risk of cardiovascular diseases in women in the United Kingdom. *JAMA Neurol.* Published online July 8, 2019. doi:10.1001/jamaneurol.2019.1812

eAppendix 1. Methods

eAppendix 2. Muscle Mass

eFigure. Muscle Mass in Patients With IIH and BMI-Matched Controls Without IIH

eTable 1. Prevalence and Incidence of IIH Among Females in the UK From 2005 to 2017

eTable 2. Incidence of IIH Among Females From 2005 to 2015 Stratified By Age At Diagnosis, BMI and Townsend Score

eTable 3. Sensitivity Analyses

This supplementary material has been provided by the authors to give readers additional information about their work.

© 2019 Adderley NJ et al. *JAMA Neurology*.

eAppendix 1. Methods

Shunt procedures

In exploratory analysis, we found that 6.5% (n = 174) of IIH patients in the CVD risk cohort had a record of shunt surgery. Among IIH patients who did and did not develop CVD, 7.4% (n = 5) and 6.5% (n = 169) respectively had had a shunt procedure; this difference was not statistically significant: p=0.77 (Chi^2 test).

© 2019 Adderley NJ et al. *JAMA Neurology*.

eAppendix 2. Muscle Mass

Previously published research by the study team has documented a similar adipose distribution in patients with IIH compared to a BMI-matched control group without IIH.[1] In the same study cohort, using the published methodology, dual-energy X-ray absorptiometry data was also generated to quantify muscle mass. This previously unpublished data found no difference in muscle mass between IIH patients and controls matched for sex, age and BMI (53.9 ± 3.7 kg in IIH versus 54.6 ± 4.3 kg in controls, p=0.6) (see eFigure 1 below). The preserved muscle mass points away from inactivity.

[1] Hornby C, Botfield H, O'Reilly MW, et al. Evaluating the Fat Distribution in Idiopathic Intracranial Hypertension Using Dual-Energy X-ray Absorptiometry Scanning. *Neuro-Ophthalmology* 2018;42:99-104. doi: 10.1080/01658107.2017.1334218

© 2019 Adderley NJ et al. *JAMA Neurology*.

eFigure. Muscle Mass in Patients With IIH and BMI-Matched Controls Without IIH (previously unpublished)

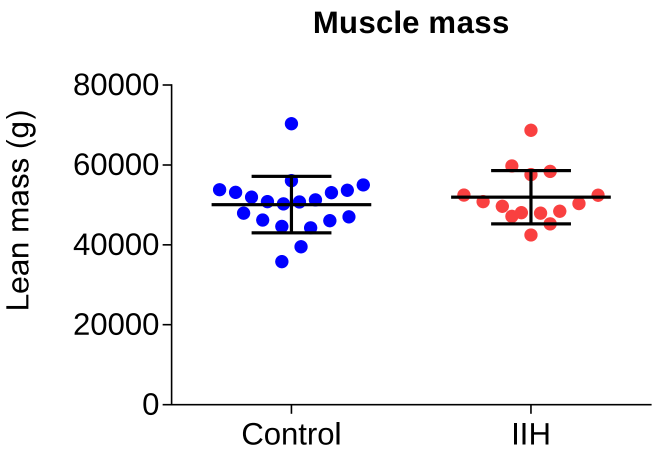

	Control	IIH	P
Age	42.2(4.3)	39.5(6.1)	0.13
BMI	34.6(3.6)	37.6(6.3)	0.11
N	20	15	

© 2019 Adderley NJ et al. *JAMA Neurology*.

eTable 1. Prevalence and Incidence of IIH among Females in the UK From 2005 to 2017

	Prevalence			Incidence		
Year	Prevalent cases	Total population	Prevalence/ 100,000 population	Incident cases	Person-years	Incidence/ 100,000 person-years
2005	520	1,986,837	26.2	52	2,082,897	2.5
2006	616	2,183,651	28.2	67	2,205,700	3.0
2007	712	2,279,040	31.2	63	2,297,936	2.7
2008	786	2,367,668	33.2	81	2,428,556	3.3
2009	936	2,533,986	36.9	94	2,543,310	3.7
2010	1036	2,566,281	40.4	101	2,534,461	4.0
2011	1135	2,563,189	44.3	106	2,569,245	4.1
2012	1249	2,618,017	47.7	120	2,654,705	4.5
2013	1382	2,673,994	51.7	150	2,598,355	5.8
2014	1417	2,523,090	56.2	166	2,447,783	6.8
2015	1504	2,356,044	63.8	153	2,165,057	7.1
2016	1394	1,967,841	70.8	172	1,856,500	9.3
2017	1397	1,768,422	79.0	152	1,642,800	9.3

© 2019 Adderley NJ et al. *JAMA Neurology*.

eTable 2. Incidence of IIH Among Females From 2005 to 2017 Stratified By Age At Diagnosis, BMI and Townsend Score

	Crude Incidence rate ratio (95% CI, p-value)	Adjusted Incidence rate ratio* (95% CI, p-value)
**BMI category**		
< 25 kg/m^2	Reference	Reference
25-30 kg/m^2	2.47 (1.96-3.11, p<0.001)	3.76 (2.98-4.74, p<0.001)
> 30 kg/m^2	11.11 (9.14-13.51, p<0.001)	17.55 (14.41-21.36, p<0.001)
**Townsend deprivation quintile**		
1 (least deprived)	Reference	Reference
2	1.00 (0.81-1.22, p=0.966)	0.92 (0.75-1.12, p=0.407)
3	1.60 (1.34-1.92, p<0.001)	1.25 (1.04-1.49, p=0.017)
4	1.68 (1.40-2.02, p<0.001)	1.15 (0.96-1.38, p=0.141)
5 (most deprived)	2.41 (2.00-2.89, p<0.001)	1.47 (1.22-1.77, p<0.001)
**Age at diagnosis**		
0-12	Reference	Reference
13-19	7.50 (5.49-10.25, p<0.001)	8.34 (6.09-11.42, p<0.001)
20-29	14.27 (10.70-19.03, p<0.001)	14.83 (10.99-20.02, p<0.001)
30-39	7.28 (5.41-9.79, p<0.001)	6.47 (4.74-8.84, p<0.001)
40-49	3.22 (2.35-4.42, p<0.001)	2.51 (1.80-3.50, p<0.001)
50-59	1.58 (1.10-2.27, p=0.014)	1.14 (0.79-1.67, p=0.481)
60-69	0.60 (0.37-0.98, p=0.043)	0.43 (0.26-0.71, p=0.001)
70 and above	0.27 (0.15-0.51, p<0.001)	0.22 (0.12-0.42, p<0.001)

*Model adjusted for age category at diagnosis, BMI category and Townsend deprivation quintile. BMI: body mass index.

© 2019 Adderley NJ et al. *JAMA Neurology*.

eTable 3. Sensitivity Analyses

	**Sensitivity Analysis 1: Incident cohort only**		**Sensitivity Analysis 2: Cohort aged below 60**	
	**Exposed**	**Unexposed**	**Exposed**	**Unexposed**
**Composite CVD**				
Population, n	1275	12421	2587	25209
Outcome events, n (%)	30 (2.30)	98 (0.78)	63 (2.38)	274 (1.08)
Person-years	5,570	52,371	12,689	125,849
Crude incidence rate (per 1000 person-years)	5.39	1.87	4.97	2.18
Follow-up years, median (IQR)	3.10 (1.28-6.21)	3.07 (1.30-6.03)	3.51 (1.34-7.11)	3.69 (1.50-7.34)
Crude HR (95% CI), p-value	2.85 (1.89-4.29); p<0.001		2.26 (1.72.-2.97.); p<0.001	
Adjusted HR (95% CI), p-value (Model 2a)	3.14 (2.07-4.78); p<0.001		2.09 (1.58-2.75); p<0.001	
**Heart failure**				
Population, n	1322	12634	2689	25704
Outcome events, n (%)	6 (0.45)	24 (0.19)	13 (0.48)	65 (0.25)
Person-years	5,726	53,249	13,220	128,627
Crude incidence rate (per 1000 person-years)	1.05	0.45	0.98	0.51
Follow-up years, median (IQR)	3.13 (1.30-6.28)	3.09 (1.31-6.08)	3.58 (1.38-7.27)	3.73 (1.51-7.42)
Crude HR (95% CI), p-value	2.30 (0.94-5.62); p=0.069		1.90 (1.05-3.44); p=0.035	
Adjusted HR (95% CI), p-value (Model 2a)	2.69 (1.07-6.79); p=0.036		1.70 (0.93-3.11); p=0.087	
**Ischaemic heart disease**				
Population, n	1310	12563	2659	25516
Outcome events, n (%)	10 (0.76)	36 (0.29)	25 (0.93)	119 (0.46)
Person-years	5,693	52,826	13,041	127,131
Crude incidence rate (per 1000 person-years)	1.76	0.68	1.92	0.94
Follow-up years, median (IQR)	3.14 (1.30-6.28)	3.08 (1.30-6.06)	3.57 (1.37-7.21)	3.70 (1.51-7.38)
Crude HR (95% CI), p-value	2.57 (1.27-5.17); p=0.008		2.03 (1.32-3.13); p=0.001	
Adjusted HR (95% CI), p-value (Model 2a)	2.81 (1.37-5.75); p=0.005		1.78 (1.15-2.76); p=0.009	
**Stroke/TIA**				
Population, n	1298	12545	2637	25527

© 2019 Adderley NJ et al. *JAMA Neurology*.

Outcome events, n (%)	16 (1.22)	53 (0.42)	38 (1.42)	142 (0.55)
Person-years	5,640	52,939	12,938	127,750
Crude incidence rate (per 1000 person-years)	2.84	1.00	2.94	1.11
Follow-up years, median (IQR)	3.10 (1.29-6.27)	3.08 (1.31-6.07)	3.51 (1.34-7.17)	3.72 (1.51-7.40)
Crude HR (95% CI), p-value	2.79 (1.59-4.87); p<0.001		2.61 (1.82-3.73); p<0.001	
Adjusted HR (95% CI), p-value (Model 2a)	3.06 (1.73-5.42); p<0.001		2.50 (1.74-3.60); p<0.001	
**Hypertension**				
Population, n	1120	11414	2214	22733
Outcome events, n (%)	66 (5.56)	307 (2.62)	145 (6.15)	957 (4.04)
Person-years	4,823	47,418	10,397	110,857
Crude incidence rate (per 1000 person-years)	13.68	6.47	13.95	8.63
Follow-up years, median (IQR)	3.00 (1.25-5.97)	2.95 (1.26-5.77)	3.19 (1.26-6.37)	3.47 (1.43-6.91)
Crude HR (95% CI), p-value	2.11 (1.61-2.75); p<0.001		1.62 (1.36-1.93); p<0.001	
Adjusted HR (95% CI), p-value (Model 2b)	1.95 (1.49-2.56); p<0.001		1.60 (1.34-1.92); p<0.001	
**Type 2 diabetes**				
Population, n	1255	11796	2471	23864
Outcome events, n (%)	34 (2.64)	264 (2.19)	120 (4.63)	700 (2.85)
Person-years	5,499	49,851	12,112	119,674
Crude incidence rate (per 1000 person-years)	6.18	5.30	9.91	5.85
Follow-up years, median (IQR)	3.11 (1.29-6.24)	3.02 (1.28-5.94)	3.49 (1.34-6.94)	3.60 (1.47-7.25)
Crude HR (95% CI), p-value	1.16 (0.81-1.66); p=0.407		1.70 (1.40-2.07); p<0.001	
Adjusted HR (95% CI), p-value (Model 2c)	0.82 (0.57-1.18); p=0.279		1.35 (1.10-1.64); p=0.003	

Incidence rates and hazard ratios for cardiometabolic outcomes among women with IIH compared to women without IIH in women with incident IIH diagnosis and in women diagnosed with IIH after the age of 60 years.
Model 2 adjusted for: age, BMI (continuous), Townsend deprivation quintile, smoking status, current (up to 65 days prior to index date) lipid-lowering drug prescription, and a. baseline hypertension and diabetes; b. baseline IHD, HF, stroke/TIA and hypertension; c. baseline IHD, HF, stroke/TIA and diabetes).
IIH: idiopathic intracranial hypertension; CVD: cardiovascular disease; TIA: transient ischaemic attack; HR hazard ratio.

© 2019 Adderley NJ et al. *JAMA Neurology*.
